# Robust Feature Selection from Microarray Data Based on Cooperative Game Theory and Qualitative Mutual Information

**DOI:** 10.1155/2016/1058305

**Published:** 2016-03-20

**Authors:** Atiyeh Mortazavi, Mohammad Hossein Moattar

**Affiliations:** ^1^Department of Computer Engineering, Imam Reza International University, Mashhad, Iran; ^2^Department of Software Engineering, Islamic Azad University, Mashhad Branch, Mashhad, Iran

## Abstract

High dimensionality of microarray data sets may lead to low efficiency and overfitting. In this paper, a multiphase cooperative game theoretic feature selection approach is proposed for microarray data classification. In the first phase, due to high dimension of microarray data sets, the features are reduced using one of the two filter-based feature selection methods, namely, mutual information and Fisher ratio. In the second phase, Shapley index is used to evaluate the power of each feature. The main innovation of the proposed approach is to employ Qualitative Mutual Information (QMI) for this purpose. The idea of Qualitative Mutual Information causes the selected features to have more stability and this stability helps to deal with the problem of data imbalance and scarcity. In the third phase, a forward selection scheme is applied which uses a scoring function to weight each feature. The performance of the proposed method is compared with other popular feature selection algorithms such as Fisher ratio, minimum redundancy maximum relevance, and previous works on cooperative game based feature selection. The average classification accuracy on eleven microarray data sets shows that the proposed method improves both average accuracy and average stability compared to other approaches.

## 1. Introduction

In feature selection, the features of the data sets are selected which are effective for predicting the target class. By eliminating additional features from data sets, the efficiency of the learning models dramatically increases. Genetic data sets have high dimensions and small size and are usually imbalanced. Increasing the high dimensions leads to classification complexity and it can reduce the classification accuracy. The small size of the data sets is another challenge [1]. Robustness issue is often ignored in feature selection. Increasing and decreasing the training samples in a nonrobust feature selection algorithm will lead to different results [2].

Feature selection methods are classified as filter methods, wrapper methods, and embedded methods [3]. Filtering methods are independent from learning algorithms. They are statistical tests which rely on the basic features of the training data and have much lower computational complexity compared with wrapper methods [4]. These methods use some measurements. Among these measurements are distance measurements [5, 6], rough set theory [7], and information theoretic measures [8]. A common distance measure is Euclidean distance, which was applied in Relief method by Kira and Rendell [5], which uses Euclidean distance to assign weights to each feature. Then, [6] developed Relief algorithm which adds the ability of dealing with multiclass problems. Peng et al. [9] proposed minimum redundancy maximum relevance (mRMR) approach, which selects features that have the highest relevance with the target class and are minimally redundant. Wang et al. [10] proposed a new filtering algorithm based on the maximum weight minimum redundancy (MWMR) criterion. The weight of the feature shows its importance and redundancy represents the correlation between the features. Nguyen et al. [11] proposed a hierarchical approach called Modified Analytic Hierarchy Process (MAHP) which uses five individual gene ranking methods including *t*-test, entropy, Receiver Operating Characteristics (ROC) curve, Wilcoxon test, and Signal to Noise Ratio (SNR) for feature ranking and selection.

In the wrapper methods, each subset is evaluated using a specific learning algorithm, and optimal features are selected to improve classification performance [3]. For example, Inza et al. [12] proposed classical wrapper algorithms for this purpose (i.e., sequential forward and backward selection, floating selection, and best-first search) and evaluated them on three microarray data sets. Another wrapper approach for gene selection from microarray data is proposed in [13] using modified ant colony optimization. Another heuristic based wrapper approach is proposed in [14] which is based on Genetic Bee Colony (GBC) optimization. More referencing materials on wrapper methods can be found in [15–18].

Similar to wrapper methods, embedded feature selection methods are classifier dependent but this relationship is stronger in embedded approaches. Guyon et al. [19] proposed Support Vector Machine (SVM) based on Recursive Feature Elimination (SVM-RFE) for feature selection in cancer classification. Canul-Reich et al. [20] introduced and applied Iterative Feature Perturbation (IFP) method, as an embedded gene selector, on four microarray data sets.

Robust feature selection algorithms are another source of researches. Yang and Mao [2] tried to improve the robustness of feature selection algorithms, with an ensemble method called Multicriterion Fusion-Based Recursive Feature Elimination (MCF-RFE). Also, Yassi and Moattar [1] presented robust and stable feature selection by integrating ranking methods and wrapper technique for genetic data classification.

The authors of [3, 21] tried to overcome the weaknesses of feature selection methods using cooperative game. They introduced a framework based on cooperative game theory to evaluate the power of each feature. To evaluate the weight of each of the features, the Banzhaf power index and the Shapley value index are used. Paper [3] used the Banzhaf power index in game theory for evaluating the weight of each feature, while paper [21] used the Shapley value index in game theory for evaluating the weight of each feature. Reference [22] presented a novel feature selection approach called Neighborhood Entropy-Based Cooperative Game Theory (NECGT) which was based on information theoretic approaches. The results of the evaluation of some UCI data sets showed that the approach yields better performance compared to classical methods in terms of accuracy.

Stability and robustness are important specially when the data is scarce and classes are imbalanced. In this paper, cooperative game theory is used for robust feature selection. In the proposed method, a cooperative game theory framework based on Shapley value is introduced for evaluating the power of each feature. To score the features in Shapley value index, we propose Qualitative Mutual Information criterion to achieve more stable results even in the presence of class imbalance and data scarcity. This criterion improves the robustness of feature selection algorithm. In this criterion, we use the Fisher ratio as the utility function in calculating Qualitative Mutual Information and this criterion leads to better robustness of the feature selection algorithm. The rest of this paper is organized as follows.  Section 2 introduces the fundamental materials and methods. In Section 3, our proposed method is described. In Section 4, the simulation results and evaluation of the proposed method are discussed. Finally, conclusions and future work are described in Section 5.

## 2. Mutual Information, Qualitative Mutual Information, and Conditional Mutual Information

The entropy, *H*(*X*), of discrete random variable *X* with *p*(*x*) = Pr(*X* = *x*) as its probability density function is defined as follows:

(1) $$\begin{array}{c} H\left(\;X\;\right)=-\underset{x\in X}{\sum }Pr\left(\;X\;\right)\log Pr\left(\;X\;\right). \end{array}$$

Also, the mutual information (MI) between two random variables *X* and *Y* with a joint probability distribution Pr(*x*, *y*) is formulated as follows [22]:

(2) MIX;Y=∑Yj∈Y ∑Xi∈XPr⁡Xi,Yjlog⁡Pr⁡Xi,YjPr⁡XiPr⁡Yj,0≤MIX;Y≤1.

Hence, Qualitative Mutual Information (QMI) is defined by multiplying a utility function *U*(*X*_*i*_, *Y*_*j*_) to mutual information formula; the formula is as follows [23, 24]:

(3) QMIX;Y=∑Yj∈Y ∑Xi∈XUXi,YjPr⁡Xi,Yj·log⁡Pr⁡Xi,YjPr⁡XiPr⁡Yj.

Different informative functions can be applied as the utility function. In the proposed approach, we use the Fisher ratio [2]. Also, Conditional Mutual Information (CMI) noted as CMI(*X*; *Y*∣*Z*) denotes the amount of information between *X* and *Y*, when *Z* is given and is formulated as follows [21]:

(4) CMIX;Y ∣ Z=∑x∈SX ∑y∈SY ∑z∈SZPr⁡X,Y,Z·log⁡Pr⁡X,Y ∣ ZPr⁡X ∣ ZPr⁡Y ∣ Z.

## 3. Proposed Approach

**Figure 1:**
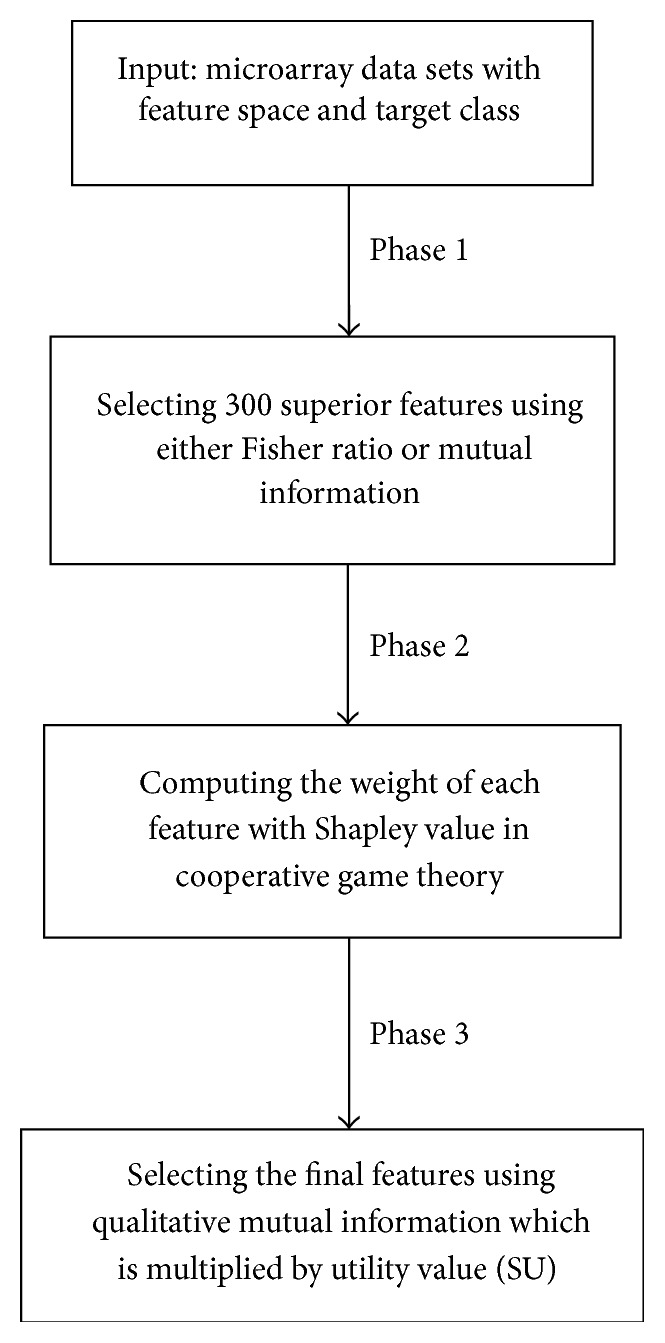
Flowchart of the proposed feature selection algorithm.

The proposed method consists of three phases, which are described as follows.  Figure 1 depicts the flowchart of the proposed feature selection algorithm.

### 3.1. Filter Approaches for Dimension Reduction

Due to high dimension of features of microarray data, in the first phase of the proposed method, the features are reduced by using one of the two filter-based feature selection methods, namely, mutual information and Fisher ratio. Fisher's ratio is an individual feature evaluation criterion. Fisher's ratio measures the discriminative power of a feature *j* by the ratio of interclass to intraclass variability. This relationship is as follows [2]:

(5) $$\begin{array}{c} FR\left(\;j\;\right)=\frac{{\left({\hat{\mu }}_{j1}-{\hat{\mu }}_{j2}\right)}^{2}}{\sigma_{j1}^{2}-\sigma_{j2}^{2}}, \end{array}$$

where ${\hat{\mu }}_{jc}$ is the sample mean of feature *j* in class *c* and *σ*_*jc*_^2^ is variance of feature *j* in *c*.

### 3.2. Feature Evaluation

In large data sets, there are intrinsic correlations among features. However, most of the filter-based feature selection algorithms discard redundant features that are highly correlated with the selected ones [21]. Each feature is weighted using information theory measures such as CMI and QMI [21].

Theoretically, the more relevancy of a feature means that it shares more information with the target class [21, 25]. Feature *f*_*i*_ is to be redundant with feature *f*_*j*_ if the following form is accepted [21]:

(6) $$\begin{array}{c} CMI\left(\;f_{j};class\;|\;f_{i}\;\right)\leq QMI\left(\;f_{j};class\;\right). \end{array}$$

Two features *f*_*i*_ and *f*_*j*_ are interdependent if the following form is accepted [21, 26]:

(7) $$\begin{array}{c} CMI\left(\;f_{j};class\;|\;f_{i}\;\right)>QMI\left(\;f_{j};class\;\right). \end{array}$$

The relevance criterion that is introduced by Peng et al. [9] is the relevance criterion of set *K* with the target class; we have

(8) $$\begin{array}{c} D\left(\;K,class\;\right)=\frac{1}{\left|K\right|}\underset{f_{j}\in K}{\sum }QMI\left(\;f_{j};class\;\right). \end{array}$$

The change of the relevance of set of *K* features with the target class considering the feature *f*_*i*_  (*f*_*i*_ ∉ *k*) which is introduced by Jain et al. [27] is measured by the following formula:

(9) CMIK;class;fi≈1K∑fj∈KCMIfj;class ∣ fi−QMIfj;class.

### 3.3. Feature Evaluation via Shapley Value

In the second phase of the proposed method, after filtering the features, the weight of each feature is obtained using the Shapley value [28, 29] for all features. In this work, we used QMI in all our formulations. The Shapley value is an efficient way for estimating the importance of features weight. The Shapley value of the *i*th feature is denoted by *ϕ*_*i*_(*v*) as is formulated as follows:

(10) $$\begin{array}{c} \phi_{i}\left(\;v\;\right)=\underset{k}{\sum }\Delta_{i}\left(\;k\;\right)\frac{\left|k\right|!\left(n-\left|k\right|-1\right)!}{n!}, \end{array}$$

where *n* denotes the total number of features and Δ_*i*_(*k*) is defined as follows:

(11) Δik=1,CMIk;class;fi≥0,  ∑fj∈Kψi,j≥K2,0,else.

**Figure 2:**
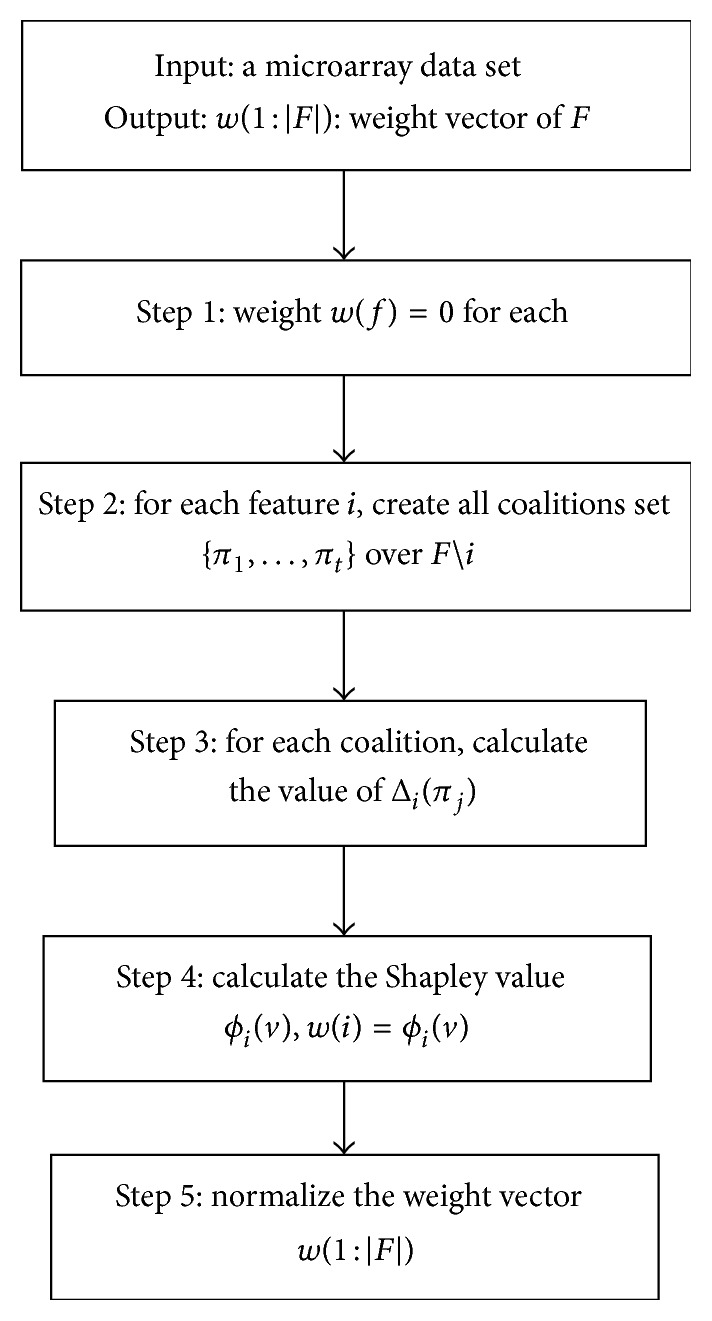
Feature evaluation flowchart.

And *ψ*(*i*, *j*) denotes the interdependence index and is defined as follows:

(12) $$\begin{array}{c} \psi \left(\;i,j\;\right)=\left\{\begin{array}{cc} 1, & CMI\left(f_{j};class|f_{i}\right)>QMI\left(f_{j};class\right),\\ 0, & else \end{array}\right. \end{array}$$

which means that the feature will be appropriate if it increases the relevance of subset *K* on the target class and is interdependent with at least half of the features [21].  Figure 2 shows the flowchart of feature evaluation. The output is a weight vector indicating the Shapley value of feature *f*_*i*_. In all the above relationships, we used QMI criterion for more robustness of selected features.

In step 2 of the proposed algorithm, for each feature *i*, we should calculate all subsets of features, so that these subsets do not contain feature *i*. So at first we calculate all two member subsets of features. Then, using (9), Conditional Mutual Information measure between set *k* and target class is calculated. Then, we calculate the Qualitative Mutual Information measure between each feature and class, namely, QMI(*f*_*j*_; class), and set these two measures in the interdependence index *ψ*(*i*, *j*). Finally, Shapley value (weight) of each feature is computed according to (10).

### 3.4. Final Feature Selection Phase

**Figure 3:**
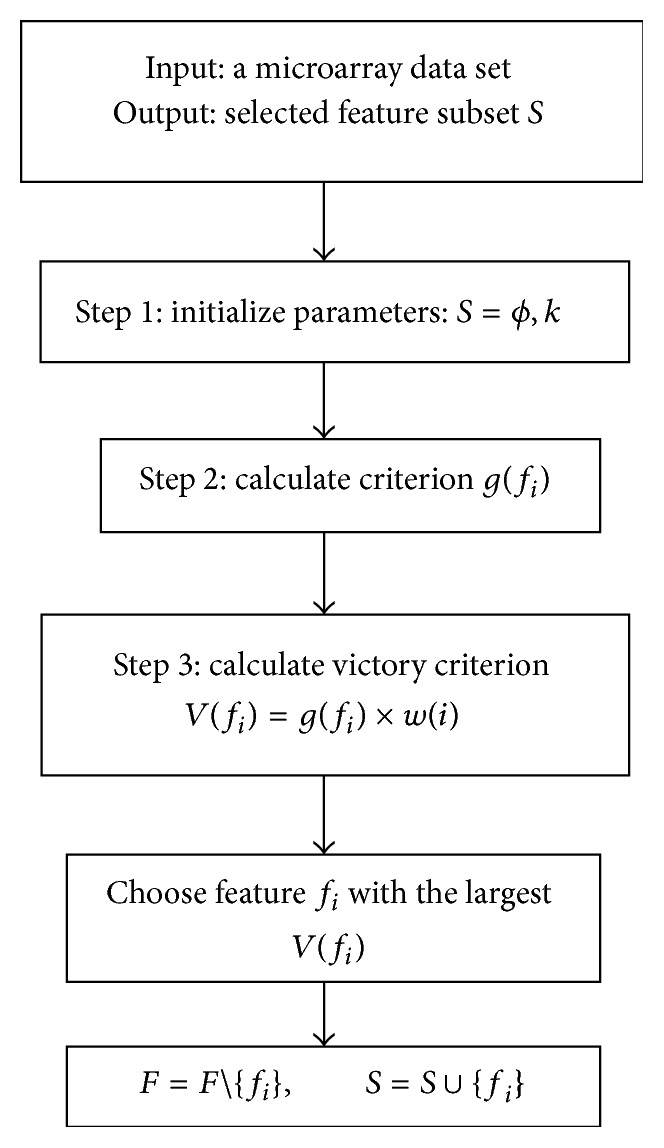
The flowchart of the final feature selection.

In the third phase that is the final phase of the proposed method, the feature selection algorithm is performed using the weight of each feature. Accordingly, we use the weights of features to reevaluate the features. For this purpose, the features with the highest weights are used. A straightforward optimization is used by employing any information criterion, such as BIF [26], SU [30], and mRMR [9]. In this paper, we used SU criterion. The formula for obtaining this criterion is provided as follows:

(13) $$\begin{array}{c} SU\left(\;f_{j},class\;\right)=2\frac{MI\left(f_{j}\;|\;class\right)}{H\left(class\right)+H\left(f_{j}\right)}, \end{array}$$

where *H* indicates the entropy and MI is the mutual information between feature and class. The flowchart of this phase is specified in Figure 3.

To select the optimal feature in each iteration, the victory criterion *V*(*f*) is defined to evaluate the superiority of each feature on others. The criterion function *g*(*f*_*i*_) (i.e., SU) is used to select feature *f*_*i*_ by feature relevance or redundancy analysis. The weight *w*(*i*), which denotes the impact of feature *f*_*i*_ on the whole feature space, is used to regulate the relative importance of evaluation value *g*(*f*_*i*_) in feature selection. Finally, we choose the features with the largest victory measure.

## 4. Experimental Results and Discussion

### 4.1. Data Set Descriptions

In this paper, we used the cancer microarray data sets from the Plymouth University [31]. For better performance and better evaluation of the proposed approach, we applied 11 high dimensional microarray data. These eleven data sets are briefly summarized in Table 1.

### 4.2. Evaluation Criteria

The criteria used to evaluate the proposed method consist of accuracy, classification precision, *F*-measure, and stability criterion for feature selection algorithm.

#### 4.2.1. Accuracy of Classification

Classification accuracy is the main criterion for evaluating the classification and predicting the samples. The classification accuracy is as follows:

(14) $$\begin{array}{c} accuracy=\frac{\left(TP+TN\right)}{\left(TP+TN+FN+FP\right)}. \end{array}$$

Accuracy is described in terms of true positives (TP), true negatives (TN), false negatives (FN), and false positives (FP).

#### 4.2.2. Precision, Recall, and *F*-Measure

The precision in (15) and recall in (16) are two other evaluation criteria. *F*-measure criterion in (17) is used to integrate precision and recall into a single criterion for the comparison:

(15) precision=TPTP+FP,

(16) recall=TPTP+FN,

(17) F-measure=2×recall×precisionrecall+precision.

#### 4.2.3. Robustness Measure

The robustness (stability) of a feature selection algorithm can be evaluated based on the ability to select repeated features, given various categories under the same distribution [2]. Let *S*_*i*_ and *S*_*o*_ denote feature subsets selected using the *i*th categories of resampled data and the full data, respectively. For measuring the similarity between two feature subsets, similarity index JC ∈ [0,1] is used which is defined as follows:

(18) $$\begin{array}{c} {JC}_{i}\left(\;k\;\right)=\frac{\left|S_{i}\cap S_{o}\right|+SC_{i}}{k}, \end{array}$$

where |*S*_*i*_∩*S*_*o*_| is the number of common features between *S*_*i*_ and *S*_*o*_, SC_*i*_ is the sum of the absolute correlation values between different features from *S*_*i*_ and *S*_*o*_, and *k* is set size of *S*_*i*_ and *S*_*o*_. Assume *m* categories of data are produced by resampling and *m* feature subsets are selected. The robustness or stability measure of the feature selection algorithm is calculated by (19) as follows:

(19) $$\begin{array}{c} \bar{JC}\left(\;k\;\right)=\frac{\sum_{i=1}^{m}{JC}_{i}\left(k\right)}{m}. \end{array}$$

### 4.3. Classification Results

First, in preprocessing phase, the features of data sets are normalized, but the SRCBT data is not normalized because its features are small. Microarray data sets are classified using* K*NN, SVM, Naive Bayes (NB), and Classification and Regression Tree (CART) classifiers. The Gaussian kernel function and standard deviation equal to 20 are used for SVM classifier in this study. We used *K* = 3 for* K*NN classifier, but since the classification result for *K* = 3 is not good for Lung_Cancer data, we used *K* = 1 for this data. Among 11 data sets, only two Prostate_Tumor and DLBCL data sets have two classes; therefore, precision and *F*-measure criteria are obtained only for these two data sets and for other data sets only the accuracy criterion is examined. The 300 superior features of 11_Tumors, 14_Tumors, Leukemia1, Leukemia2, and DLBCL data sets are selected using mutual information criterion. For other data sets, Fisher ratio is applied for prior feature selection.

**Figure 4:**
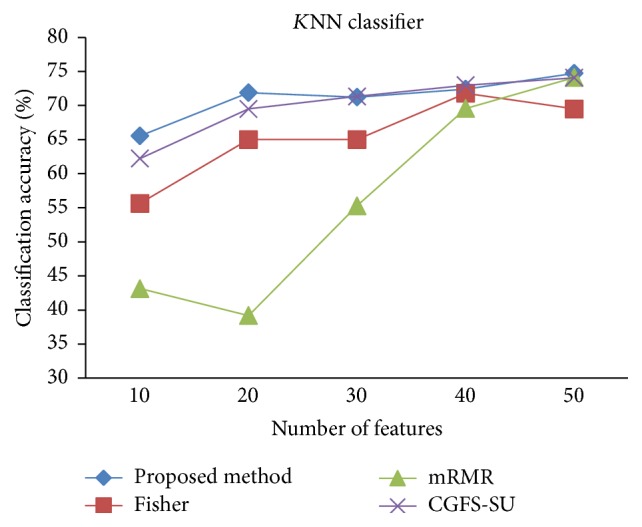
*K*NN classification accuracy for 11_Tumors.

**Figure 5 fig5:**
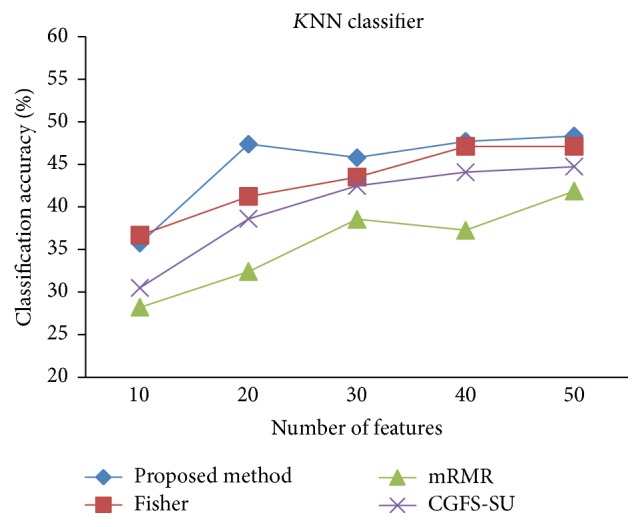
*K*NN classification accuracy for 14_Tumors.

**Figure 6 fig6:**
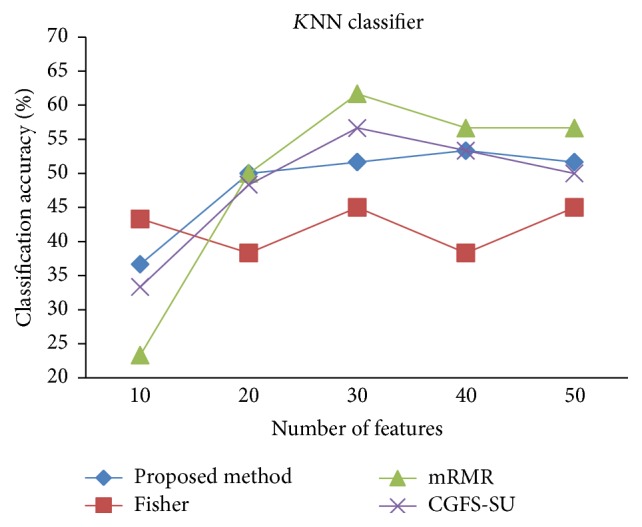
*K*NN classification accuracy for 9_Tumors.

**Figure 7 fig7:**
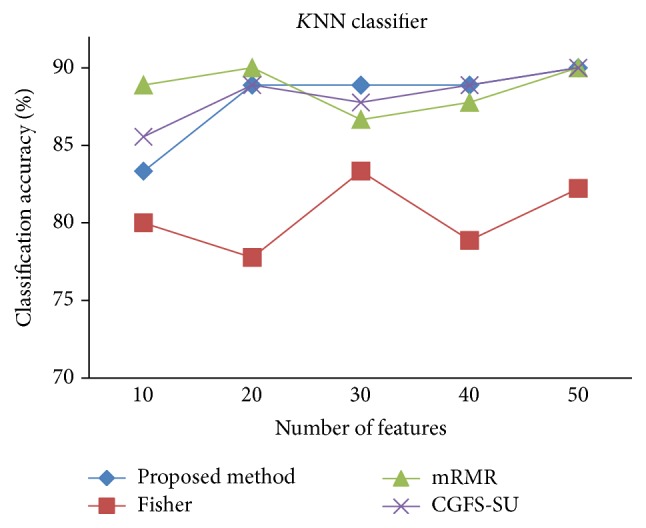
*K*NN classification accuracy for Brain_Tumor1.

**Figure 8 fig8:**
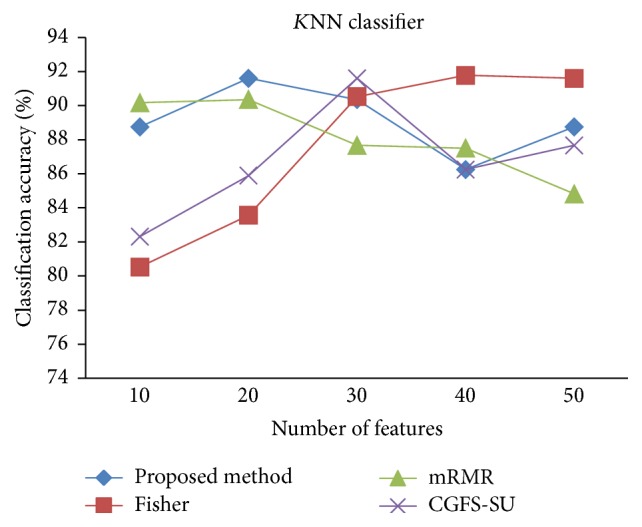
*K*NN classification accuracy for Leukemia1.

**Figure 9 fig9:**
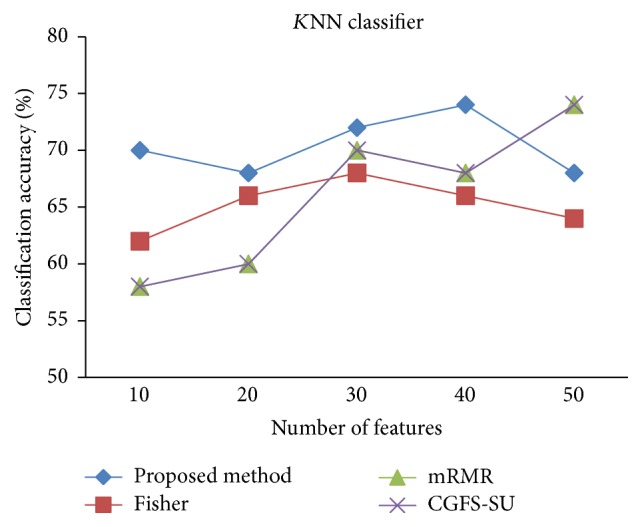
*K*NN classification accuracy for Brain_Tumor2.

**Figure 10 fig10:**
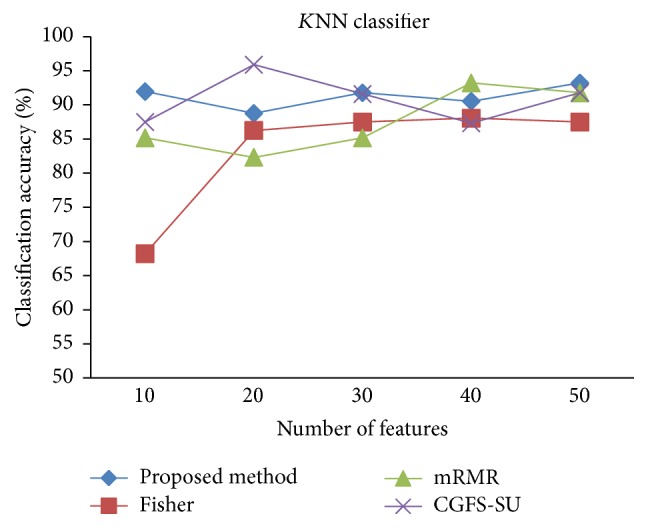
*K*NN classification accuracy for Leukemia2.

**Figure 11 fig11:**
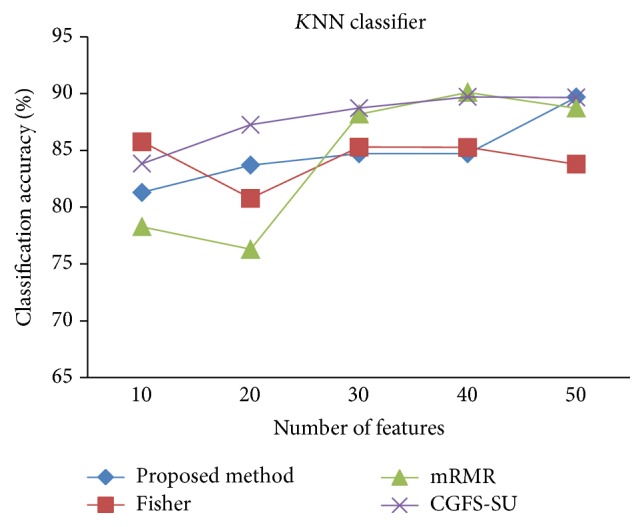
*K*NN classification accuracy for Lung _Cancer.

**Figure 12 fig12:**
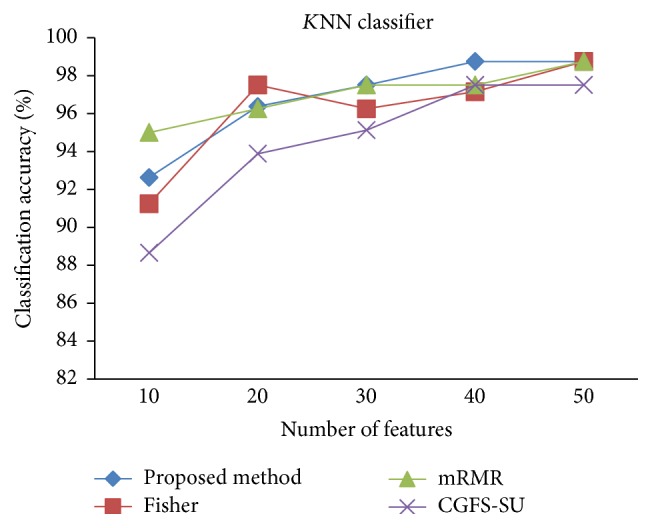
*K*NN classification accuracy for SRBCT.

**Figure 13 fig13:**
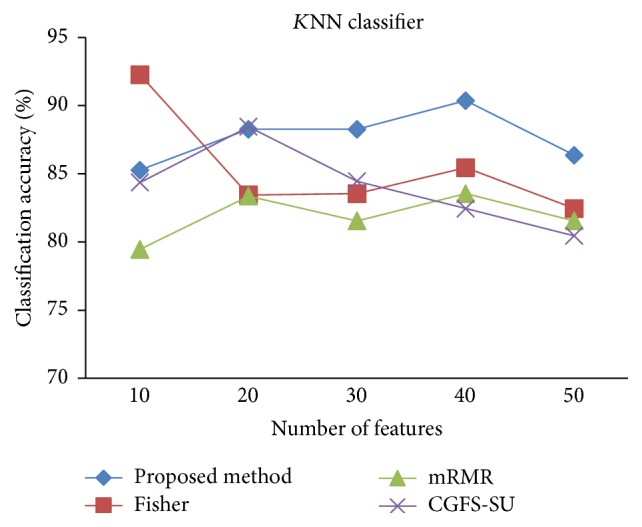
*K*NN classification accuracy for Prostate_Tumor.

**Figure 14 fig14:**
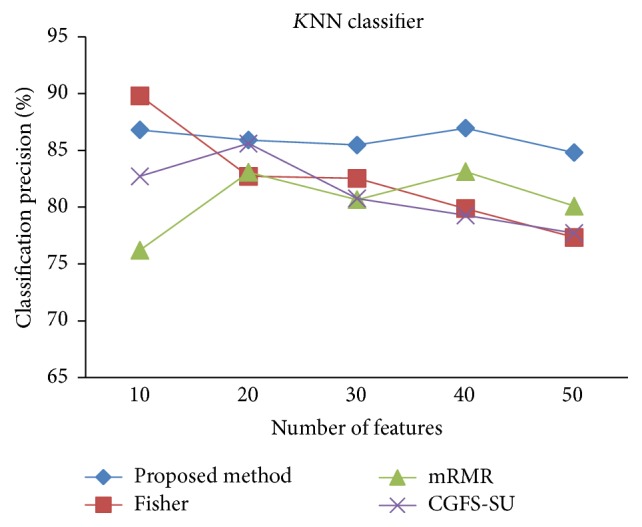
*K*NN classification precision for Prostate_Tumor.

**Figure 15 fig15:**
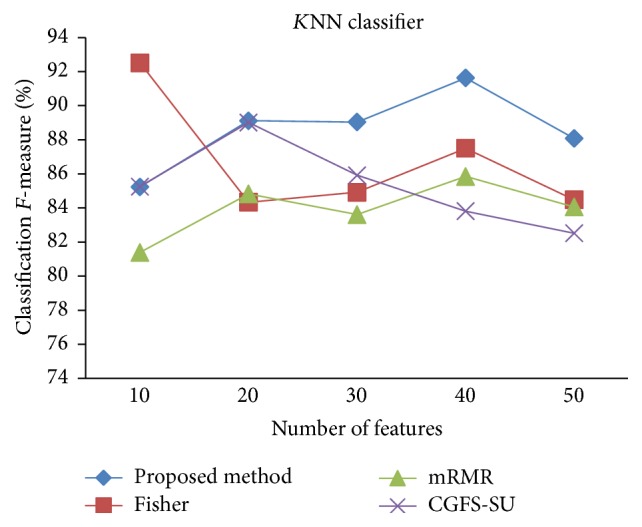
*K*NN classification *F*-measure for Prostate_Tumor.

**Figure 16 fig16:**
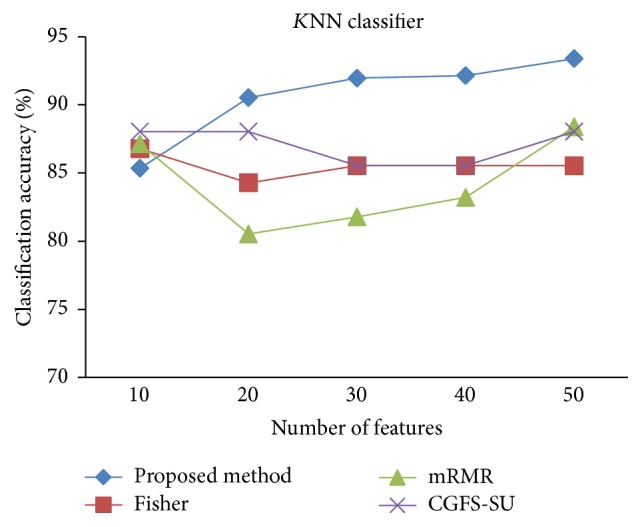
*K*NN classification accuracy for DLBCL.

**Figure 17 fig17:**
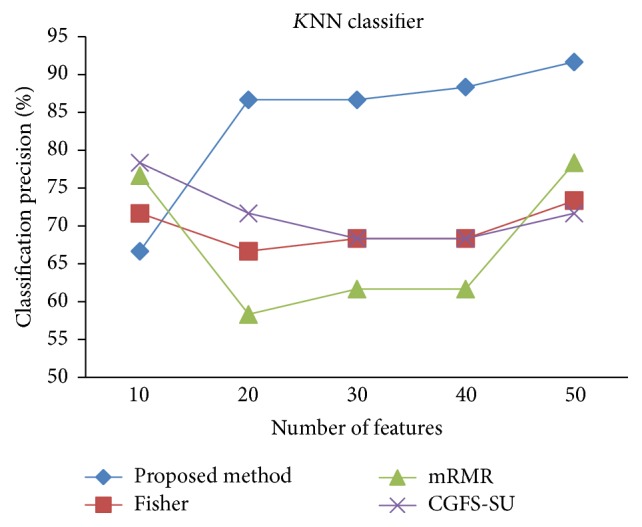
*K*NN classification precision for DLBCL.

**Figure 18:**
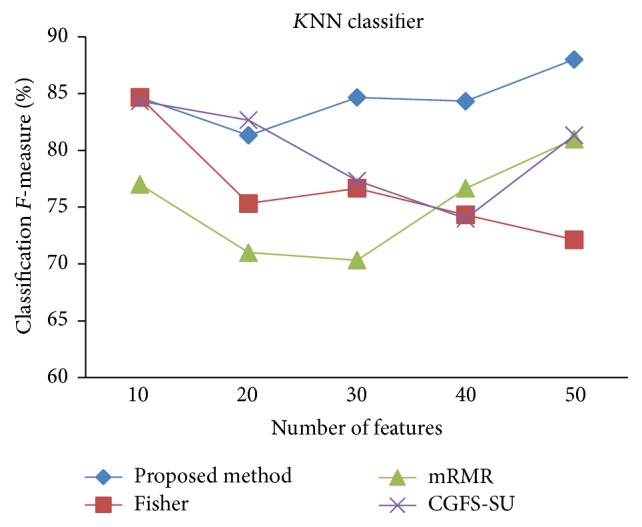
*K*NN classification *F*-measure for DLBCL.

For comparison, the classification accuracy is given against the number of features. The *x*-axis is the number of features which is considered up to 50 and the *y*-axis is the classification accuracy. The proposed method is compared with feature selection using Fisher's ratio, mRMR, and CGFS-SU. The mRMR algorithm is used as information criterion algorithm for comparison. Fisher's ratio method is a univariate filter method for evaluating each feature individually and the CGFS-SU method is proposed in [21] for cooperative game based feature selection. For estimating the performance of classification algorithms, tenfold cross-validation is used. Figures 4 [Fig fig5] [Fig fig6] [Fig fig7] [Fig fig8] [Fig fig9] [Fig fig10] [Fig fig11] [Fig fig12] [Fig fig13] [Fig fig14] [Fig fig15] [Fig fig16] [Fig fig17]–18 show the classification results for eleven data sets using* K*NN classifier.

*K*NN classifier results show that the proposed method has been improved in most cases compared to the other methods of feature selection. For 11_Tumors data, the proposed method has achieved the maximum accuracy of 74.73 percent. Also, the result of CGFS-SU method is almost the same as the proposed method and is 74.08 percent, but in 30 first features, its accuracy is less than the proposed method. In 14_Tumors data, the results are low for all feature selection algorithms. However, the proposed method has the maximum accuracy of 48.34 percent and other methods have much lower accuracy, which is due to the weak correlation between selected features. However, it can be observed that the proposed method achieves higher accuracy in all 50 features as compared to other methods.

**Figure 19:**
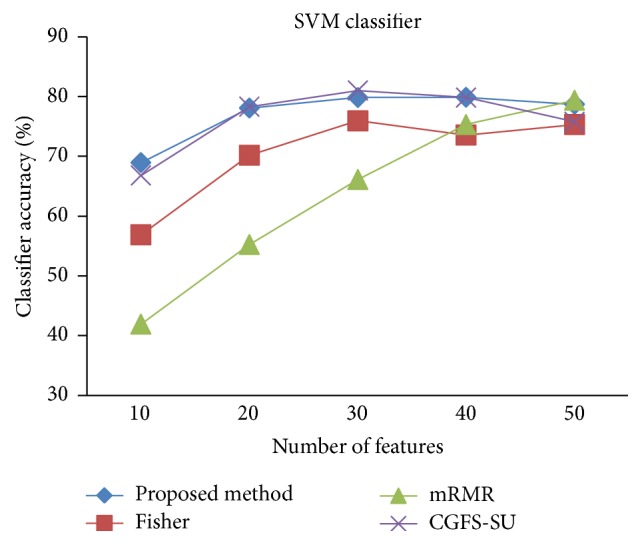
SVM classification accuracy for 11_Tumors.

**Figure 20 fig20:**
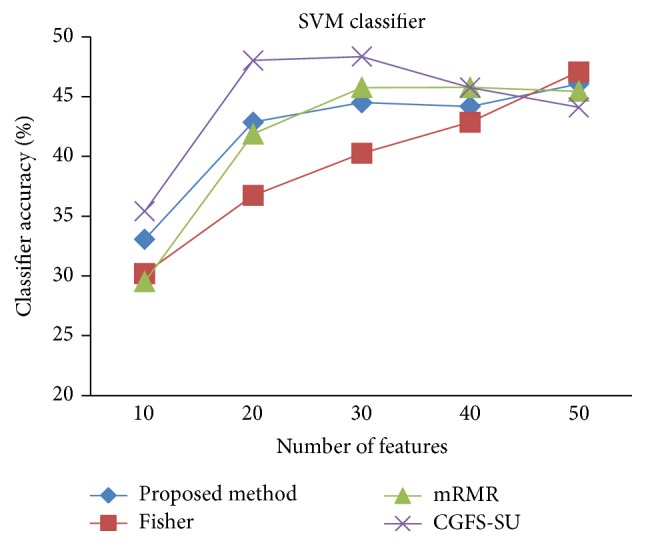
SVM classification accuracy for 14_Tumors.

**Figure 21 fig21:**
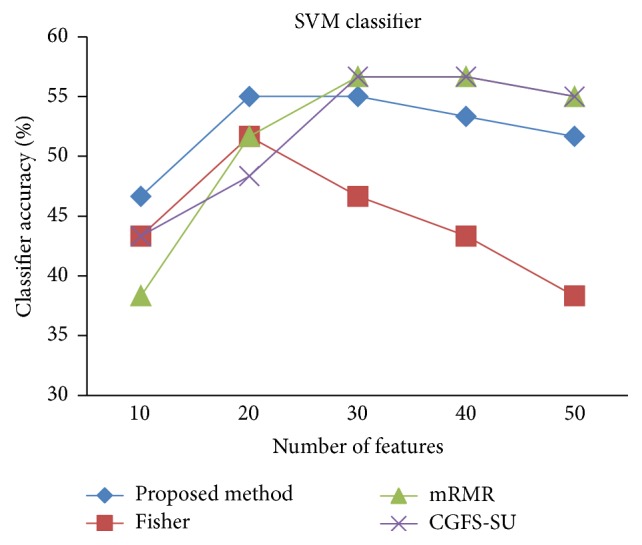
SVM classification accuracy for 9_Tumors.

**Figure 22 fig22:**
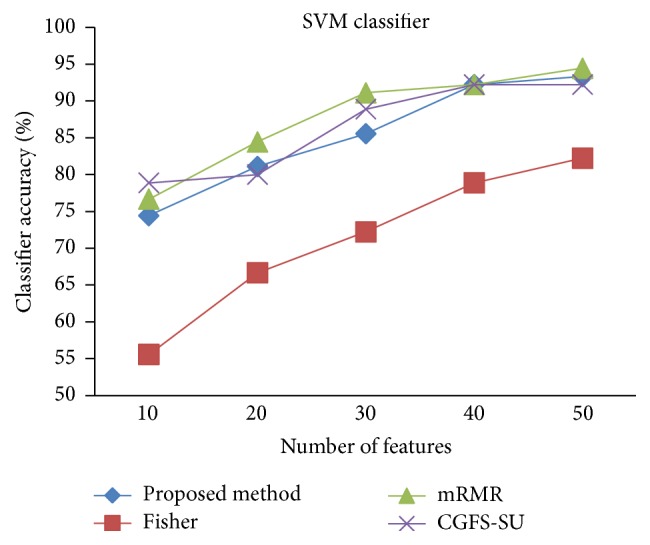
SVM classification accuracy for Brain_Tumor1.

**Figure 23 fig23:**
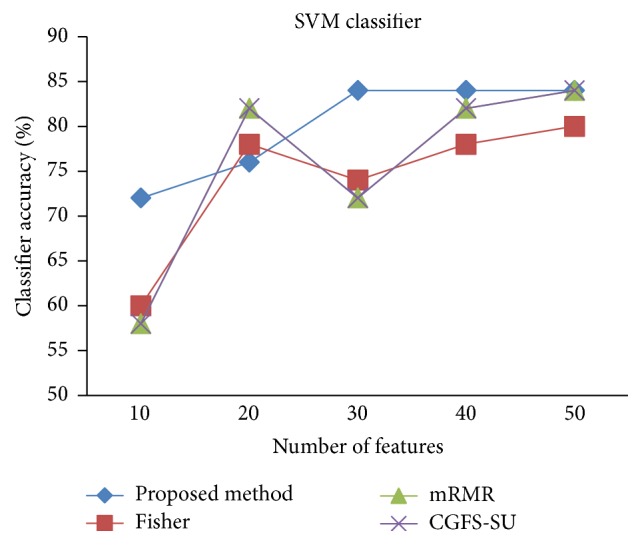
SVM classification accuracy for Brain_Tumor2.

**Figure 24 fig24:**
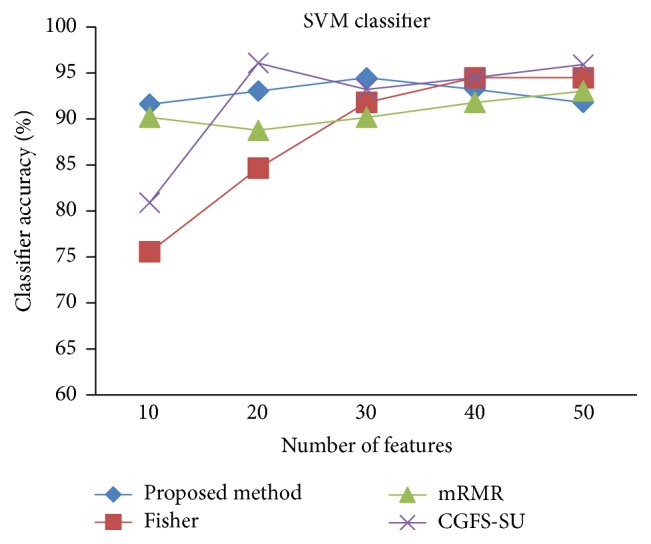
SVM classification accuracy for Leukemia1.

**Figure 25 fig25:**
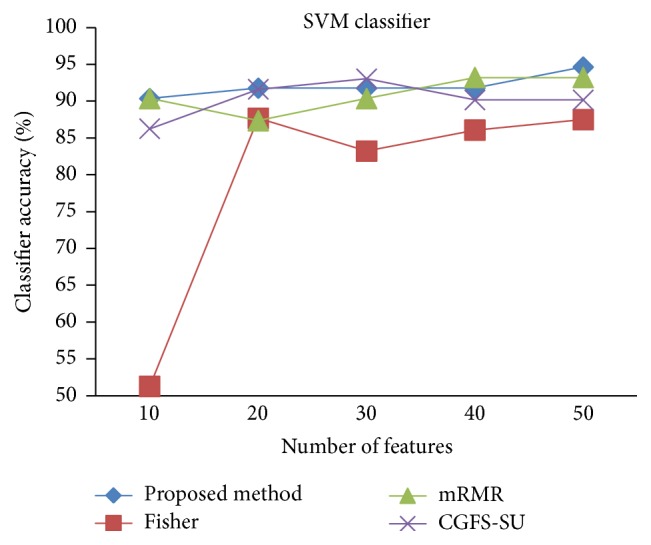
SVM classification accuracy for Leukemia2.

**Figure 26 fig26:**
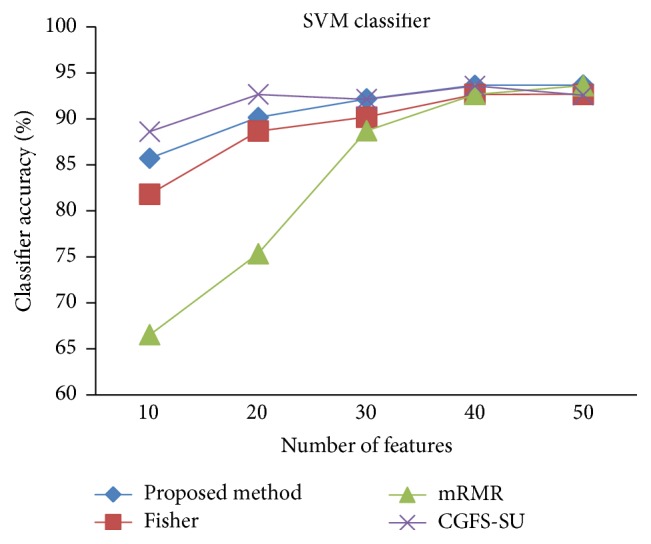
SVM classification accuracy for Lung_Cancer.

**Figure 27 fig27:**
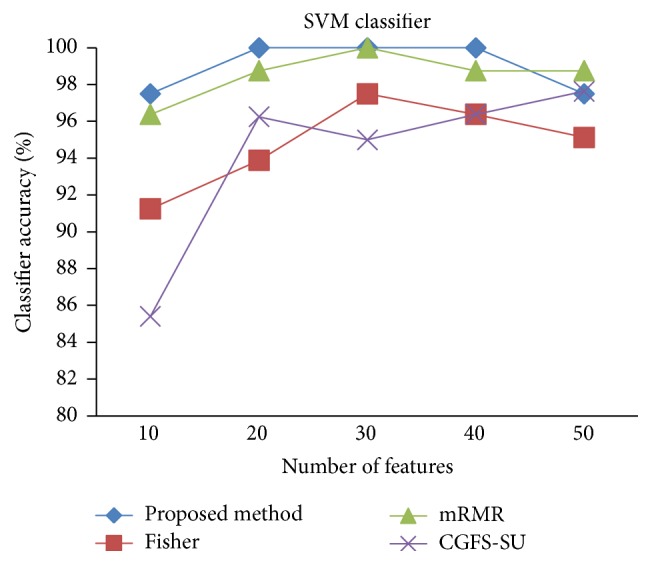
SVM classification accuracy for SRBCT.

**Figure 28 fig28:**
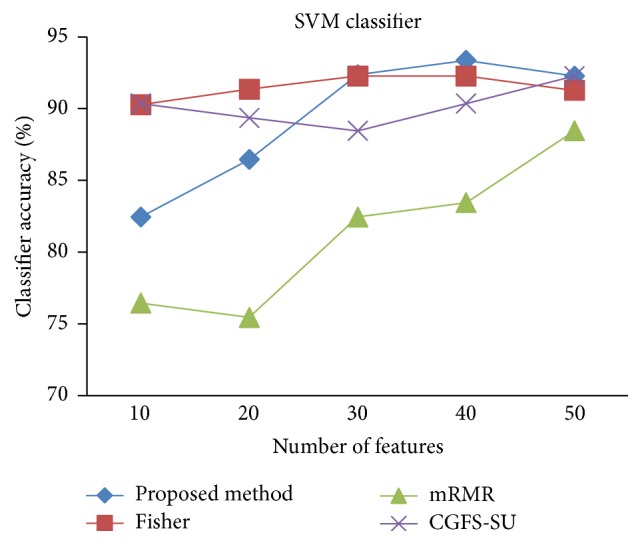
SVM classification accuracy for Prostate_Tumor.

**Figure 29 fig29:**
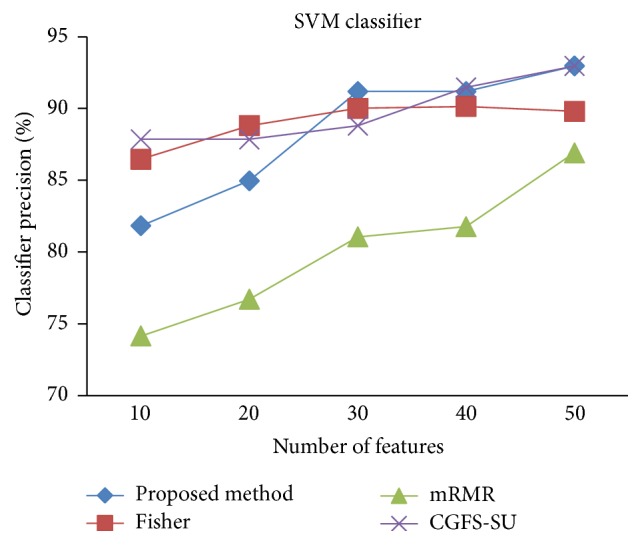
SVM classification precision for Prostate_Tumor.

**Figure 30 fig30:**
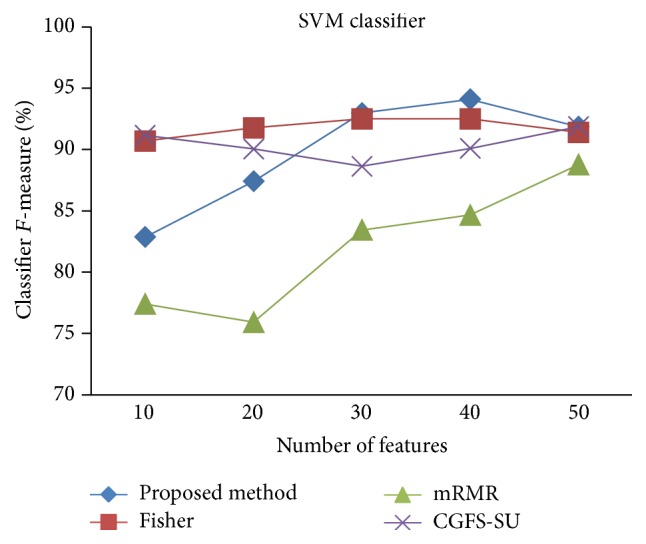
SVM classification *F*-measure for Prostate_Tumor.

**Figure 31 fig31:**
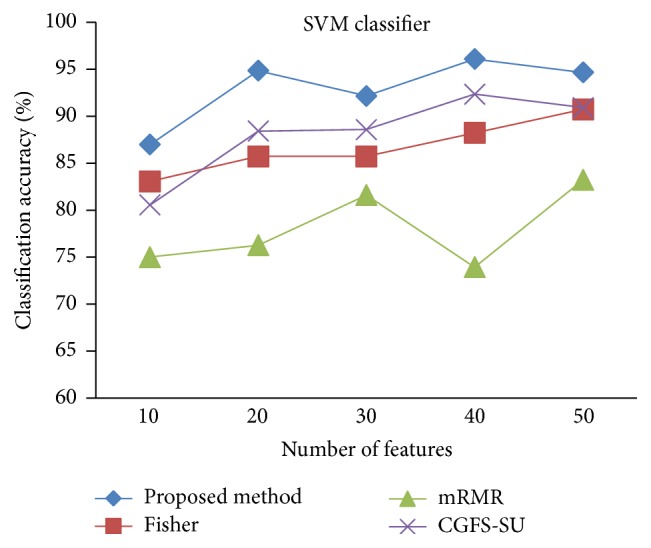
SVM classification accuracy for DLBCL.

**Figure 32 fig32:**
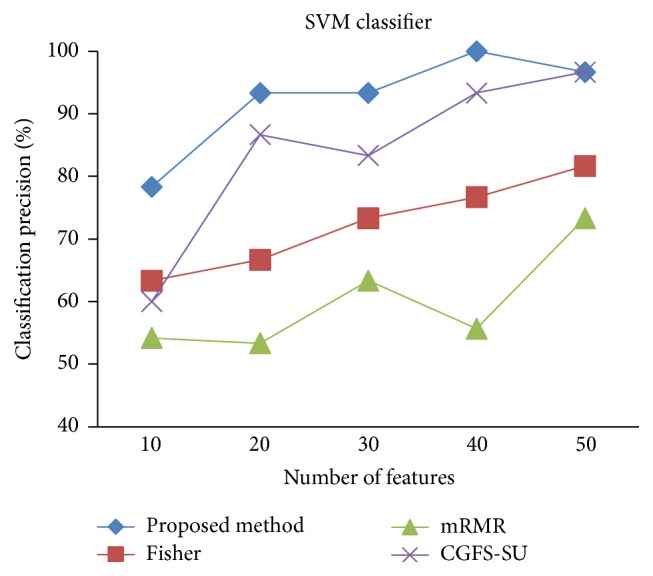
SVM classification precision for DLBCL.

**Figure 33:**
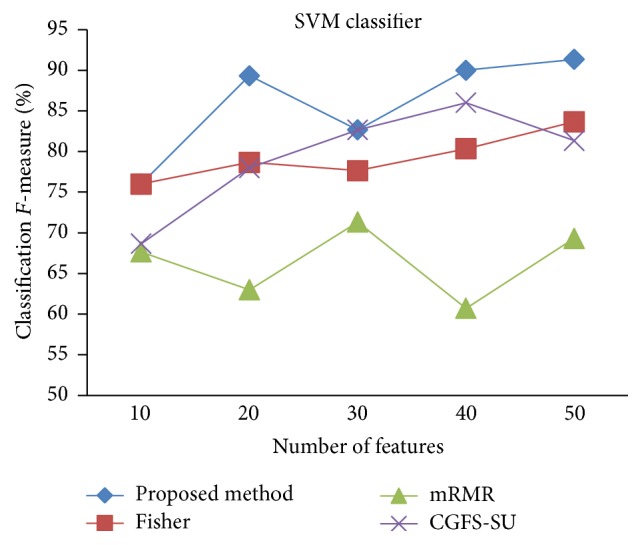
SVM classification *F*-measure for DLBCL.

The accuracy has been increased in SRBCT data, and the proposed method and Fisher and mRMR algorithms reached the maximum accuracy of 98.75 percent. Furthermore, the CGFS-SU method accuracy is 97.56 percent. In Prostate_Tumors data, it has been shown that the proposed method achieved maximum accuracy among all feature selection methods for all three evaluation criteria and for all cases. For DLBCL data, the proposed method achieved the maximum value for most cases for each of the three criteria, and it reached the highest accuracy of 93 percent, precision of 91 percent, and *F*-measure of 88 percent compared to other methods. This shows that the proposed method could reduce interdependency between groups of features and is effective as compared with other algorithms. Figures 19 [Fig fig20] [Fig fig21] [Fig fig22] [Fig fig23] [Fig fig24] [Fig fig25] [Fig fig26] [Fig fig27] [Fig fig28] [Fig fig29] [Fig fig30] [Fig fig31] [Fig fig32]–33 show the classification results using SVM classifier on the evaluation data sets.

The results of most data sets with SVM classifier are better compared to* K*NN classifier. In SRBCT data, the proposed method reached the highest rate of accuracy of 100 percent for 10 to 40 features and also the mRMR method. But the CGFS-SU method has lower accuracy and the maximum accuracy is 97.63 percent. Also, Fisher criterion is not much accurate and its maximum accuracy is 97.5 percent. It may be due to the fact that the relationship between the features and target classes is maximum for this data set and the mRMR method and the proposed approach have been able to retain this relationship between features. Also, in DLBCL data, the proposed method is superior to the other approaches for all three criteria.

### 4.4. Stability Results

**Figure 34:**
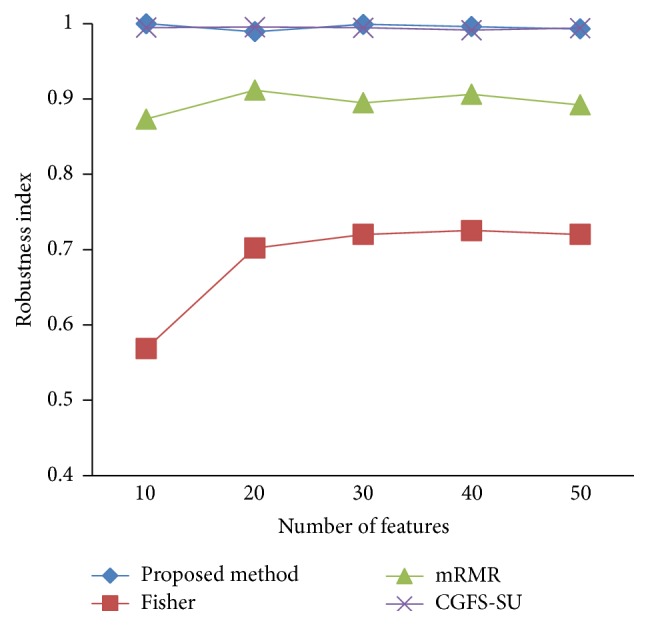
Stability results for 11-Tumors.

**Figure 35 fig35:**
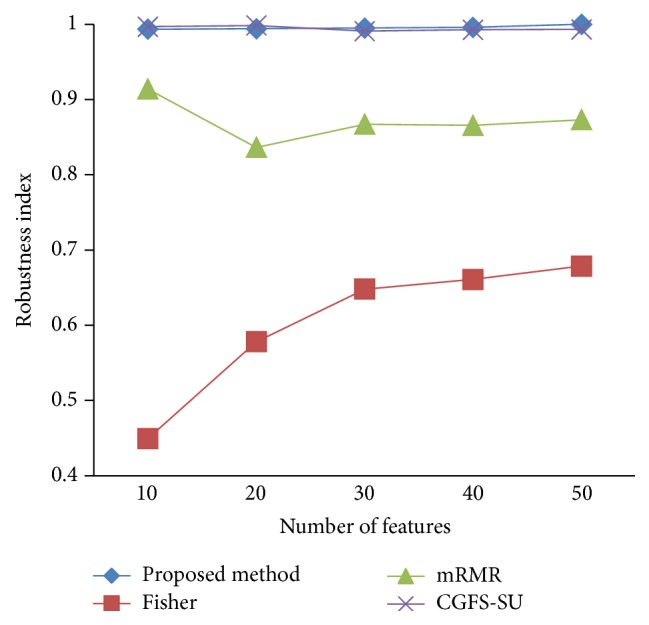
Stability results for 14_Tumors.

**Figure 36 fig36:**
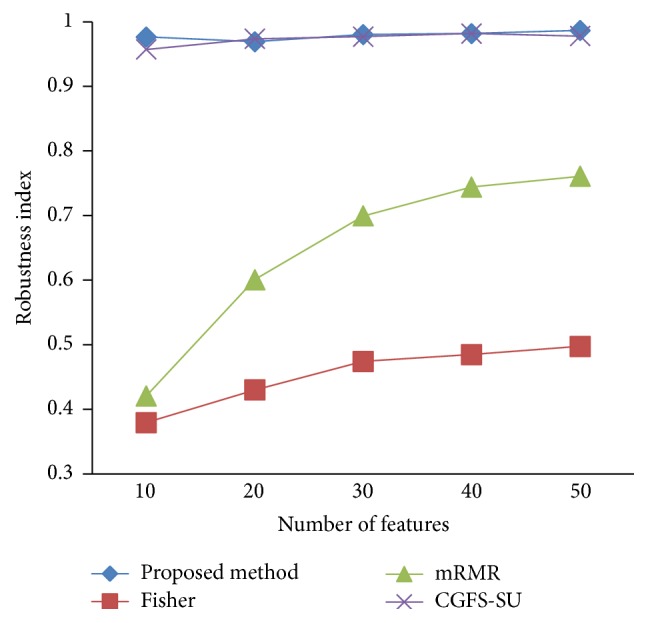
Stability results for 9_Tumors.

**Figure 37 fig37:**
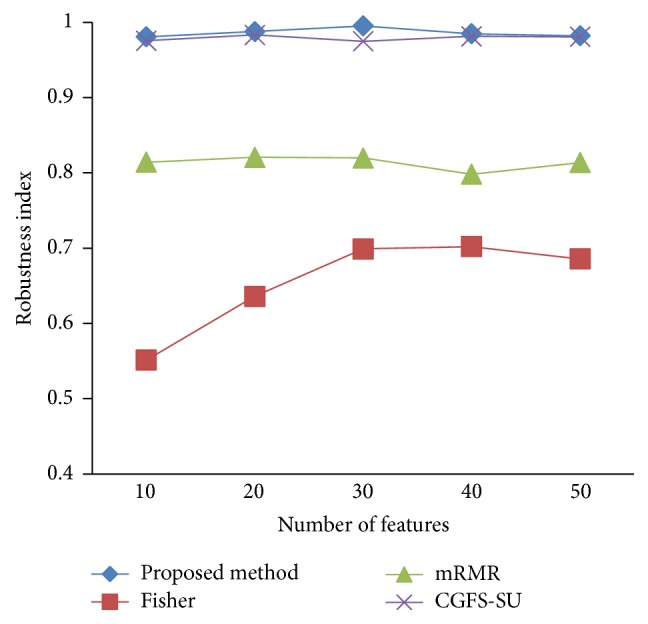
Stability results Brain_Tumor1.

**Figure 38 fig38:**
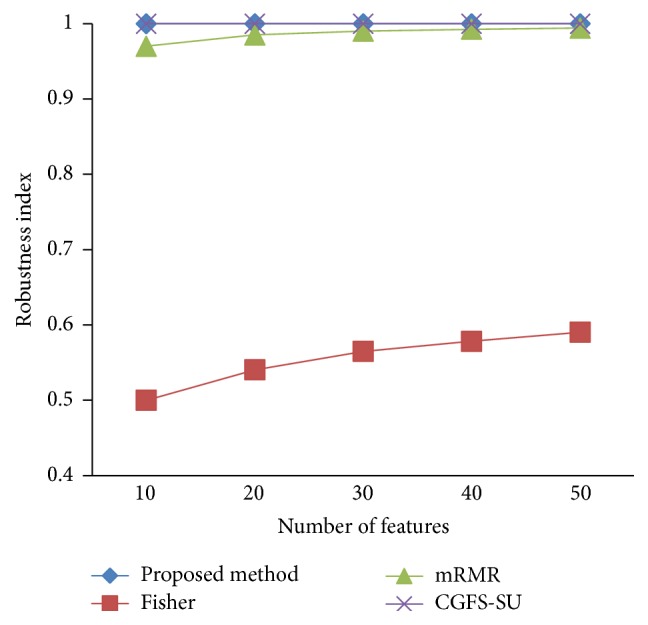
Stability results for Brain_Tumor2.

**Figure 39 fig39:**
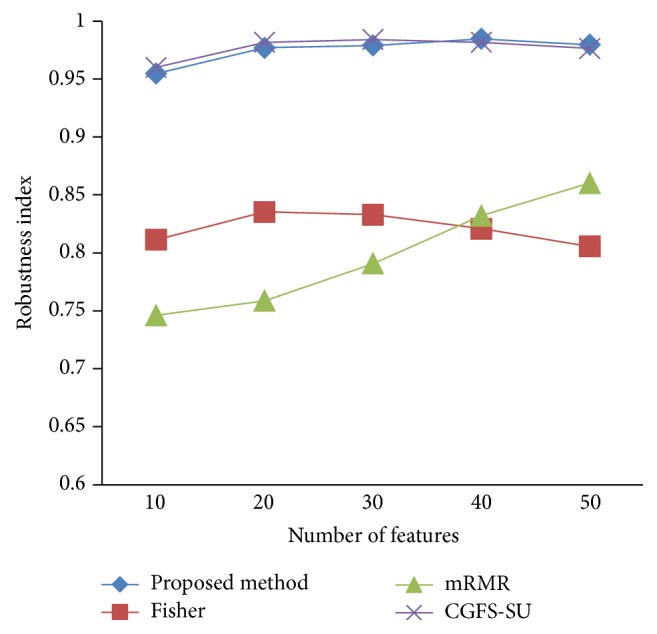
Stability results for Leukemia1.

**Figure 40 fig40:**
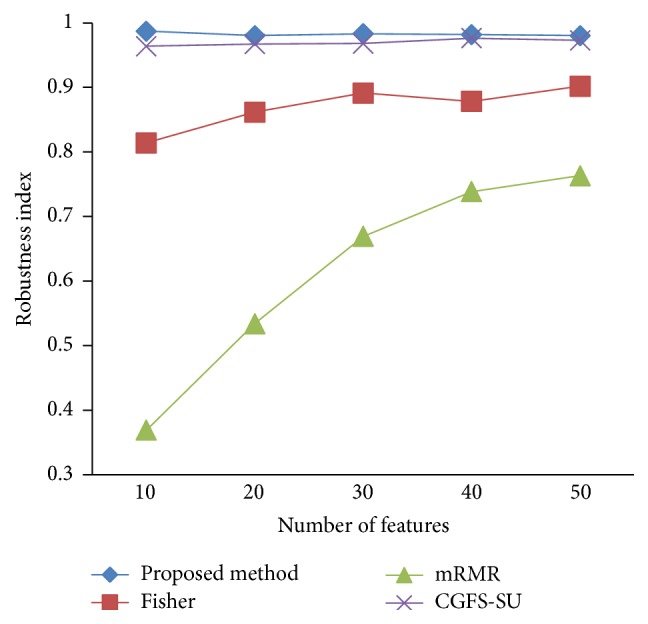
Stability results for Leukemia2.

**Figure 41 fig41:**
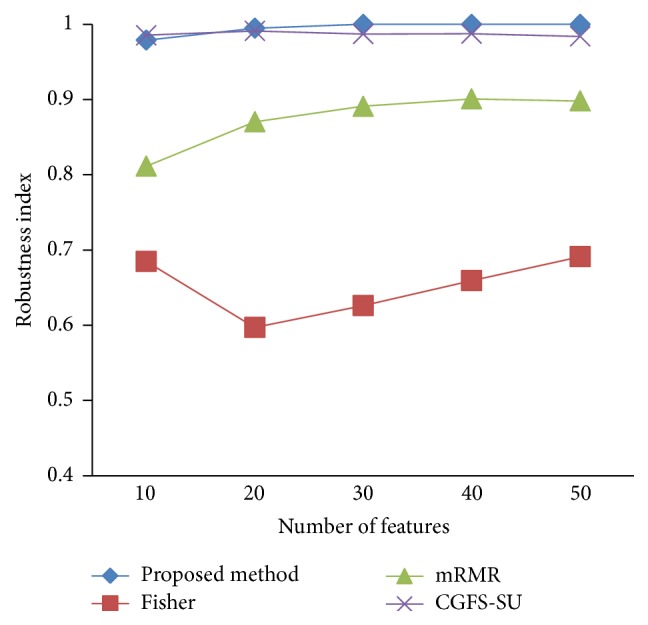
Stability results for Lung_Cancer.

**Figure 42 fig42:**
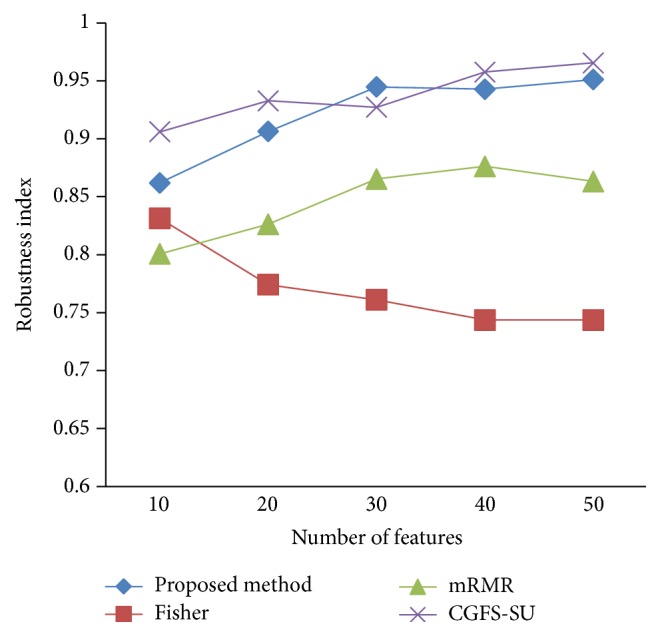
Stability results for SRCBT.

**Figure 43 fig43:**
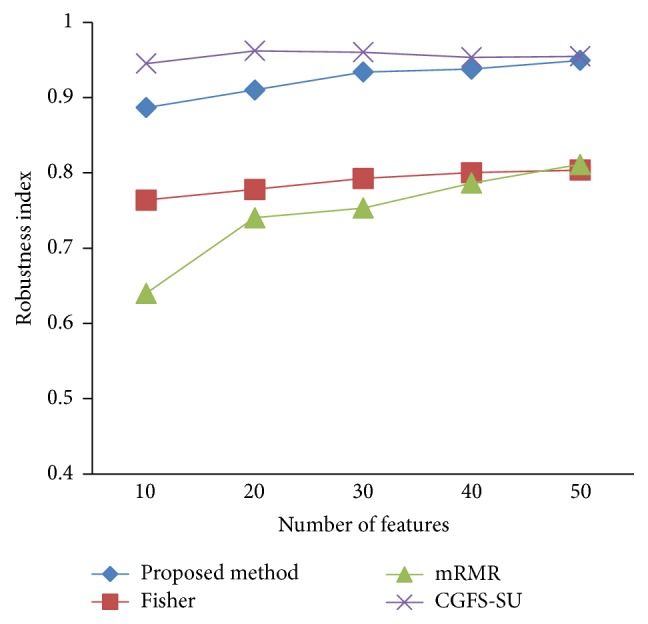
Stability results for Prostate_Tumor.

**Figure 44:**
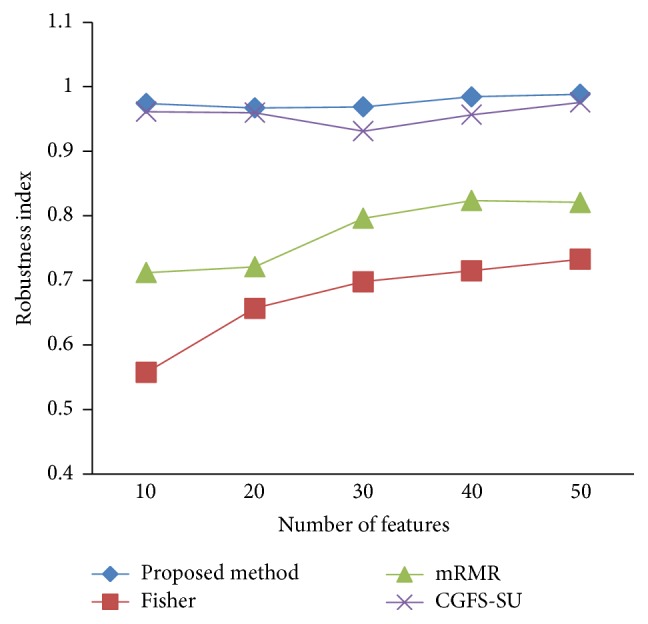
Stability results for DLBCL.

In the stability diagram, the *x*-axis shows the number of features and the *y*-axis shows the stability index. As denoted above, the stability index value is between 0 and 1. For this criterion, features are normalized except for the SRCBT data. Fisher ratio is applied to select the prior 300 best features of all data sets. For estimating the stability, we used tenfold cross-validation. The results of stability are observed in Figures 34 [Fig fig35] [Fig fig36] [Fig fig37] [Fig fig38] [Fig fig39] [Fig fig40] [Fig fig41] [Fig fig42] [Fig fig43]–44.

The stability values of the CGFS-SU and the proposed methods are very close to each other and are slightly different. But we have seen that the proposed method reached the maximum stability in 11_Tumors, 14_Tumors, Brain_Tumor2, and Lung_Cancer data sets. This shows that the proposed method is much more robust than the other feature selection approaches. However, the CGFS-SU method achieved the maximum stability only for Brain_Tumor2 data. The stability of other feature selection methods has significant differences with the CGFS-SU and the proposed method; that is, the stability of mRMR method is approximately equal to these methods only for Brain_Tumor2 data.  Table 2 shows the maximum accuracy and average accuracy of feature selection methods on evaluation microarray data using* K*NN, SVM, NB, and CART classifiers.

According to Table 2, it can be seen that the proposed method achieved the highest accuracy among all the algorithms in most cases. Also, results of the average classification accuracy show that the proposed method has improved the average accuracy between 1 and 5 percent as compared with the CGFS-SU method, also between 2 and 14 percent as compared with the Fisher ratio method, and between 1 and 14 percent as compared with the minimum redundancy maximum relevance approach. In Table 3, average stability** ±** standard deviation of four feature selection methods is shown.

In these experiments, if the average stability is high and the standard deviation is low, the approach is more robust. It is observed that, among the eleven microarray data sets, the stability of the proposed method is more than the other feature selection methods on eight data sets and the stability is less than the CGFS-SU method only on three data sets. Furthermore, the results of these experiments show that the proposed method has improved the average stability between 0.001 and 0.01 as compared with the CGFS-SU method, between 0.1 and 0.5 as compared with the Fisher ratio method, and between 0.01 and 0.3 as compared with the minimum redundancy maximum relevance method.

## 5. Conclusion and Future Work

This paper proposed a feature selection approach for robust gene selection of microarray data. In the proposed method, a cooperative game theory method is introduced to evaluate the weight of each feature considering the complex and inherent relationships among features and Qualitative Mutual Information (QMI) measure is used for more robust feature selection. The idea used for the stability is to use Fisher ratio as a utility in calculating QMI. The results on eleven evaluation microarray data sets show that the proposed method is an effective and stable method for reducing the dimensions of data and is able to reach relative improvement as compared to the other feature selection methods. As future works, we propose to calculate the weight of each feature using fuzzy Shapley value or fuzzy Banzhaf power index.

## Figures and Tables

**Table 1:** Characteristics of the evaluation data sets.

Number	Data set	Features	Samples	Classes
1	11_Tumors	12533	174	11
2	14_Tumors	15009	308	26
3	9_Tumors	5726	60	9
4	Brain_Tumor1	5920	90	5
5	Brain_Tumor2	10367	50	4
6	Leukemia1	5327	72	3
7	Leukemia2	11225	72	3
8	Lung_Cancer	12601	203	5
9	SRBCT	2309	83	4
10	Prostate_Tumor	12600	102	2
11	DLBCL	7129	77	2

**Table 2:** Maximum values of accuracy and average accuracy of feature selection methods on eleven microarray data sets.

Data set	Classifier	Accuracy	Proposed approach	CGFS-SU	Fisher	mRMR
11_Tumors	*K*NN	Max	**74.73**	74.08	71.79	74.11
		Average	**71.16**	70.02	65.39	56.26
	SVM	Max	79.86	**81.01**	75.94	79.37
		Average	**77.07**	76.34	70.36	63.59
	Naive Bayes	Max	**84.96**	82.67	77.58	83.16
		Average	74.75	**77.62**	69.83	72.46
	DecisionTree	Max	**64.37**	62.05	60.35	61.07
		Average	**61.64**	60.20	56.05	50.33

14_Tumors	*K*NN	Max	**48.34**	44.73	47.11	41.86
		Average	**44.99**	40.08	43.13	35.66
	SVM	Max	46.07	**48.35**	47.06	45.76
		Average	42.13	**44.33**	39.42	41.67
	Naive Bayes	Max	**45.22**	41.18	40.92	37.23
		Average	43.87	40.19	**59.8**	37.04
	DecisionTree	Max	**46.05**	43.81	41.25	37.98
		Average	**41.67**	39.90	37.98	33.76

9_Tumors	*K*NN	Max	53.33	56.66	45	**61.66**
		Average	48.66	48.33	41.99	**49.46**
	SVM	Max	55	**56.66**	51.66	**56.66**
		Average	**52.33**	52	44.66	51.66
	Naive Bayes	Max	53.33	**56.18**	51.8	48.32
		Average	**52.87**	51.9	50.35	46.90
	DecisionTree	Max	**50**	48.33	41.66	46.66
		Average	**42**	40.66	37	**42**

Brain_Tumor1	*K*NN	Max	**90**	**90**	83.33	**90**
		Average	88	88.22	80.44	**88.66**
	SVM	Max	93.33	92.22	82.22	**94.44**
		Average	85.33	86.44	71.11	**87.77**
	Naive Bayes	Max	**85.55**	82.22	**85.55**	84.44
		Average	**83.77**	81.11	81.11	82.44
	DecisionTree	Max	**78.44**	71.11	72.44	70.22
		Average	**80**	73.33	74.44	72.22

Brain_Tumor2	*K*NN	Max	**74**	**74**	68	**74**
		Average	**70.4**	66	65.2	66
	SVM	Max	**84**	**84**	80	**84**
		Average	**80**	75.6	74	75.6
	Naive Bayes	Max	**82**	80	68	80
		Average	**73.2**	68.8	63.2	68.8
	DecisionTree	Max	**60**	56	**60**	54
		Average	50	52.40	**53.20**	50.40

Leukemia1	*K*NN	Max	91.60	90.35	**91.78**	91.60
		Average	**89.14**	86.75	87.60	88.10
	SVM	Max	93.21	**96.07**	94.46	93.03
		Average	**92.82**	92.10	88.17	90.78
	Naive Bayes	Max	**97.32**	**97.32**	94.64	97.14
		Average	94.46	**95.89**	92.89	95.28
	DecisionTree	Max	**91.78**	87.85	87.67	90.35
		Average	**90.32**	85	81.82	88.71

Leukemia2	*K*NN	Max	93.21	**95.89**	87.50	93.21
		Average	**91.25**	90.82	83.10	87.53
	SVM	Max	**94.64**	93.03	87.67	93.21
		Average	**92.07**	90.25	79.14	90.99
	Naive Bayes	Max	**70.71**	69.28	51.07	59.64
		Average	**69.89**	64.39	47.67	54.89
	DecisionTree	Max	88.75	**90.17**	85.89	87.67
		Average	**86.53**	86.03	77.60	84.21

Lung_Cancer	*K*NN	Max	89.69	89.66	85.76	**90.11**
		Average	84.82	**87.84**	84.17	84.32
	SVM	Max	**93.66**	93.57	92.66	93.61
		Average	91.06	**91.90**	89.19	83.36
	Naive Bayes	Max	96.09	**96.57**	91.69	66.07
		Average	92.35	**92.55**	90.57	60.12
	DecisionTree	Max	**88.16**	86.28	83.28	87.26
		Average	**85.42**	85.17	81.86	79.46

SRCBT	*K*NN	Max	**98.75**	97.56	**98.75**	**98.75**
		Average	**96.80**	94.13	96.25	97
	SVM	Max	**100**	97.63	97.5	**100**
		Average	**99**	94.13	94.88	98.52
	Naive Bayes	Max	98.75	98.75	**100**	98.75
		Average	**97.27**	95.33	94.88	95.75
	DecisionTree	Max	87.91	85.41	**89.16**	88.05
		Average	82.55	83.55	**85.08**	80.08

Prostate_Tumor	*K*NN	Max	90.36	84.45	**92.27**	83.54
		Average	**87.70**	84.03	85.43	81.89
	SVM	Max	**93.36**	92.27	92.27	88.45
		Average	89.38	90.14	**91.49**	81.25
	Naive Bayes	Max	**91.27**	85.45	82.45	79.45
		Average	**90.12**	82.03	80.85	77.45
	DecisionTree	Max	**91.27**	80.36	82.36	76.27
		Average	**87.07**	78.36	78.92	73.41

DLBCL	*K*NN	Max	**93.39**	88.03	86.78	88.39
		Average	**90.67**	86.03	85.53	84.21
	SVM	Max	**96.07**	92.32	90.71	83.21
		Average	**92.92**	88.14	86.67	78
	Naive Bayes	Max	91.96	**93.57**	84.46	83.03
		Average	87.10	**90.64**	83.21	79.71
	DecisionTree	Max	**83.21**	81.78	82.58	81.78
		Average	**81.39**	78.60	80.78	78.53

**Table 3:** Average stability ± standard deviation stability of the evaluated feature selection approaches.

Data set	Proposed method	CGFS-SU	Fisher	mRMR
11_Tumors	**0.9954 ± 0.0044**	0.9939 ± 0.0015	0.6872 ± 0.0668	0.8956 ± 0.0149
14_Tumors	**0.9956 ± 0.0026**	0.9944 ± 0.0030	0.6029 ± 0.0940	0.8712 ± 0.0279
9_Tumors	**0.9790 ± 0.0065**	0.9736 ± 0.0096	0.4557 ± 0.0472	0.6451 ± 0.1400
Brain_Tumor1	**0.9873 ± 0.0053**	0.9790 ± 0.0037	0.6548 ± 0.0636	0.8132 ± 0.0090
Brain_Tumor2	**1 ± 0**	**1 ± 0**	0.5548 ± 0.0358	0.9863 ± 0.0097
Leukemia1	0.9750 ± 0.0117	**0.9768 ± 0.0098**	0.8212 ± 0.0130	0.7975 ± 0.0483
Leukemia2	**0.9826 ± 0.0029**	0.9697 ± 0.0048	0.8695 ± 0.0345	0.6203 ± 0.1538
Lung_Cancer	**0.9947 ± 0.0092**	0.9869 ± 0.0027	0.6517 ± 0.0398	0.8743 ± 0.0372
SRCBT	0.9214 ± 0.0375	**0.9378 ± 0.0240**	0.7707 ± 0.0362	0.8463 ± 0.0317
Prostate_Tumor	0.9235 ± 0.0250	**0.9551 ± 0.0066**	0.7876 ± 0.0165	0.7462 ± 0.0655
DLBCL	**0.9766 ± 0.0094**	0.9569 ± 0.0161	0.6719 ± 0.0700	0.7748 ± 0.0543
